# Therapeutic targeting of post-translational modifications in ovarian cancer: mechanisms and clinical applications

**DOI:** 10.1186/s13048-025-01833-w

**Published:** 2025-11-11

**Authors:** Ling Ke, Ying Zhang

**Affiliations:** 1 Department of Obstetrics and Gynecology, Affiliated Hospital of Guangdong Medical University, Zhanjiang, 524001 China; 2 Department of Obstetrics and Gynecology, Maoming People’s Hospital, Maoming, 525000 China

**Keywords:** Post-translational modifications, Ovarian cancer, Pathogenesis, Therapeutic strategies

## Abstract

Ovarian cancer is a lethal disease with high mortality due to late diagnosis, recurrence, and chemotherapy resistance. There is an urgent need for better diagnostic and therapeutic strategies. Recently, protein post-translational modifications (PTMs) have gained significant attention for their role in the onset, progression, and treatment of ovarian cancer. PTMs (including phosphorylation, ubiquitination, methylation, ADP-ribosylation, and others) significantly influence tumor cell proliferation, metastasis, and drug resistance by modulating cellular signal transduction, DNA repair mechanisms, and metabolic processes. PARP inhibitors block the active site of PARP1 in *BRCA*-mutant ovarian cancer, disrupting ADP-ribosylation, inducing apoptosis in cancer cells, and extending progression-free survival. However, the emergence of drug resistance, such as *BRCA* reversal mutations, and the insufficient specificity of targets remain significant limitations to therapeutic efficacy. Strategies targeting other modification pathways, such as ubiquitination and methylation, provide promising options for expanding treatments. Combination therapies, like integrating PARP inhibitors with chemotherapy or immunotherapy, and precision medicine also show potential to overcome therapeutic challenges. This article systematically examines the pivotal role of PTMs in ovarian cancer pathogenesis, outlines therapeutic strategies and associated challenges in targeting PTMs, and offers a comprehensive overview with strategic guidance for future research.

## Introduction

Ovarian cancer (OC) is one of the most common gynecological malignancies, with a 5-year survival rate of only about 30% [1]. The World Cancer Observatory estimates that 207,252 people died from ovarian cancer worldwide in 2020. In particular, high-grade serous carcinoma (HGSC) is the most common and lethal subtype of OC, accounting for 70–80% of deaths. HGSC is characterized by asymptomatic tumor growth and is diagnosed at an advanced stage of cancer metastasis [2]. At the time of diagnosis, ovarian cancer often spreads to pelvic organs due to invasion and metastasis of ovarian cancer cells through peritoneal fluid or ascites [3]. For patients with ovarian cancer, the first-choice treatment option is to undergo primary cytoreductive surgery to achieve an R0 status, followed by chemotherapy. For cancers where R0 resection is not achievable, neoadjuvant chemotherapy is needed for treatment to achieve complete or partial remission of the disease. Paclitaxel combined with carboplatin still serves as the first-line chemotherapy regimen for ovarian cancer. Nevertheless, the recurrence rate after first-line treatment of ovarian cancer is as high as 75% [4]. Secondly, approximately 75% of ovarian cancer patients experience recurrence due to their own or acquired chemotherapy resistance, resulting in cancer recurrence [5]. Post-translational modification dysfunction is correlated with adverse prognosis and chemotherapy resistance, and has been reported in multiple types of cancers.

Protein post-translational modifications (PTMs), including phosphorylation, ubiquitination, methylation, acetylation, glycosylation, etc., is a very important biological process [6]. Phosphorylation takes place at specific amino acids, typically at serine, threonine, or tyrosine residues [7]. The advancement of the cell cycle is controlled by the phosphorylation and dephosphorylation of substrates of cyclin-dependent kinases (CDKs) [8]. Histones and nonhistone proteins methylation can take place at lysine or arginine residues, executed by protein lysine and arginine methyltransferases (PKMTs and PRMTs) [9]. Histone methylation is essential in managing chromatin assembly and gene regulation, impacting pathological processes across various cancers [10]. The regulation of protein acetylation is achieved through the combined actions of lysine acetyltransferases (KATs) and lysine deacetylases (KDACs), which respectively add and remove acetyl groups from lysine residues, and participate in many processes such as metabolism and signal transduction [11]. Due to its importance in physiological and pathological processes, p53 activity is tightly regulated by multiple mechanisms, especially post-translational modifications. Acetylation is a key modification mechanism for p53 activation in stress response [12]. The process of ADP-ribosylation is facilitated by enzymes known as ADP-ribosyltransferases (ARTs), including the PARP family, which plays an important role in DNA damage repair [13]. The FDA has approved three PARP inhibitors—olaparib, rucaparib, and niraparib—for treating ovarian cancer [14]. Dysregulation of ubiquitination and deubiquitination has been noted in several cancer types [15].

In recent years, with the rapid development of proteomics technologies, such as high-precision mass spectrometry analysis that can accurately identify modification sites and types, and bioinformatics tools that can deeply mine modification-related data, post-translational modification research has become increasingly in-depth and has attracted much attention in the field of oncology. Post-translational modifications in tumor cells are often disordered, laying the groundwork for tumor occurrence, development, metastasis, and drug resistance, while also opening up new avenues for anti-cancer treatment. As a result, numerous drugs targeting post-translational modification enzymes or substrates have emerged and are expected to become new weapons in the fight against cancer. Due to the complexity of ovarian cancer, studying post-translational modifications is crucial for overcoming this challenging disease.

## Investigation into the correlation between the pathogenesis of ovarian cancer and Post-translational modifications

### Pathogenic factors of ovarian cancer

#### Genetic factors play a dominant role

Genetic factors play a crucial role in the development of ovarian cancer. Approximately 10–15% of women with epithelial ovarian cancer carry a *BRCA1* or *BRCA2* mutation [16]. These two genes are tumor suppressor genes. The proteins encoded by them are involved in the repair of DNA double-strand breaks through the homologous Recombination pathway, governing DNA damage response, regulating DNA transcription and chromosome recombination, and suppressing tumors through inducing apoptosis and other means [17, 18]. Defects in the DNA repair pathways, such as mutations in *BRCA1* and *BRCA2*, can enhance the PARP1 activity in breast and ovarian cancers. Moreover, PARP inhibitor therapy can specifically eliminate tumor cells containing *BRCA1* and *BRCA2* gene mutations [19, 20]. Consequently, the inhibition of PARPs has emerged as a therapeutic strategy aimed at *BRCA1/2*-mutated cancer cells. Importantly, *BRCA* mutations underlie hereditary breast and ovarian cancer (HBOC), and women who carry *BRCA-1* pathogenic variants (mutations) have a cumulative lifetime risk of breast cancer of up to 72% and ovarian cancer of up to 72% (44%) [21]. Apart from the *BRCA* genes, mutations in genes like *p53*, *PTEN*, *AMT*, *RAD50*, *BRIP1*, and *PALB2* can also increase the risk of ovarian cancer to varying extents. For example, a mutation in the *p53* gene can disrupt cell cycle regulation and apoptosis induction, causing abnormal cell proliferation [22, 23]. Consequently, it is evident that genetic factors exert a profound influence on the onset of ovarian cancer.

#### Hormones and environmental inducing factors

Ovarian cancer is a complex disease influenced by various factors, including hormonal regulation. Estrogen was shown to drive the epithelial–mesenchymal transition (EMT) in many tissues [24]. Epithelial-mesenchymal transition has been extensively documented as a critical process implicated in the dissemination and invasive behavior of ovarian cancer cells [25]. Estrogen signaling is mediated by estrogen receptors (ERs), which include classical ERα, ERβ, and non-classical GPER1. In the development of ovarian cancer, the expression of ERα is increased, and the expression of ERβ is decreased, and the imbalance of the two may play a key role [26]. Given the critical role of the estrogen receptor signaling pathway in ovarian cancer development, a novel estrogen-targeted polyethylene glycol liposome (ES-SSL-OXA/PTX) encapsulating oxaliplatin and paclitaxel was developed to target the highly expressed ERs on the surface of SKOV-3 cells. This formulation aims to enhance therapeutic efficacy while minimizing side effects in SKOV-3 tumor treatment, representing a promising approach for future clinical management of ovarian cancer [27]. In addition, the findings of a study demonstrated that estrogen diminishes PTEN expression levels via the ESR1 genomic pathway and phosphorylates PTEN through the GPR30-PKC nongenomic pathway, consequently activating the PI3K/AKT/mTOR signaling cascade [28]. This dual mechanism plays a critical role in determining the fate of epithelial ovarian cancer (EOC) cells.

### The key role of post-translational modification

#### Phosphorylation

Barnali Deb et al. [29]. identified differentially regulated phosphopeptide signatures at 880 phosphorylation sites in five cancer types (breast, colon, LUAD, ovarian, and UCEC). Insulin receptor substrate 4 (IRS4), part of the insulin receptor substrate family, acts as a cytoplasmic adaptor protein, relaying signals from receptor protein tyrosine kinases. It is an oncogenic driver in cancers like breast cancer and non-small cell lung cancer (NSCLC). In breast cancer, IRS4 fosters tumor growth and resistance to HER2-targeted therapy by persistently activating the PI3K/AKT pathway. This hyperactivation is due to the absence of a SHP2-binding domain, crucial for therapy resistance [30]. In NSCLC, IRS4 is overexpressed, promoting cancer progression via the PI3K/Akt and Ras-MAPK pathways and leading to resistance against EGFR-TKIs like gefitinib. Reducing IRS4 can restore drug sensitivity, suggesting that targeting IRS4 may help overcome resistance in NSCLC treatment [31]. The PI3K/AKT pathway is crucial in cancer therapy-related multidrug resistance, as its abnormal activation affects targets like apoptosis proteins and ABC transporters, inhibiting apoptosis and enhancing cell survival. Thus, targeting this pathway, possibly through IRS4 modulation, could help counteract therapeutic resistance in cancer [32]. A recent study has demonstrated that FER is markedly upregulated in ovarian cancer and subsequently activates the downstream PI3K- AKT signaling pathway through the phosphorylation of IRS4. FER recognizes and binds to IRS4, subsequently adding phosphate groups to specific tyrosine residues. Following phosphorylation, the affinity between IRS4 and the PI3K regulatory subunit PIK3R2 markedly increases, thereby promoting sustained activation of the PI3K-AKT signaling pathway and providing a continuous supply of proliferation signals for cancer cells. Furthermore, higher FER expression levels are associated with poorer prognoses in ovarian cancer patients, underscoring its significance as a potential therapeutic target [33]. Enhanced ERK phosphorylation signifies the activation of the MAPK signaling pathway [34]. In ovarian cancer, growth factors, hormones, and other stimuli often induce the activation of Ras protein. This activation subsequently leads to the phosphorylation and activation of Raf kinase, which in turn phosphorylates and activates MEK. Ultimately, MEK phosphorylates ERK [35]. Activated ERK 1/2 enters the nucleus and phosphorylates several transcription factors, including c-Fos, c-Myc, and c-Jun, thereby promoting cell growth, survival, and proliferation [36]. Furthermore, members of the protein kinase C (PKC) family are frequently aberrantly activated in ovarian cancer. PKCα can enhance the migratory and invasive capabilities of cancer cells by phosphorylating downstream target proteins [37]. Specifically, it can phosphorylate adhesion molecules to reduce intercellular adhesion, thereby facilitating the escape of cancer cells from the primary tumor site and infiltration into surrounding tissues [38]. Additionally, PKCα acts on cytoskeletal proteins to assist in cancer cell deformation and movement, ultimately promoting metastasis [39]. A study highlights PKCα’s crucial role in the mesenchymal-to-amoeboid transition, which boosts cancer cell invasiveness. Activating PKCα maintains amoeboid morphology, while inhibiting it shifts cells to a less invasive mesenchymal form. This suggests PKCα is key in cancer invasiveness and a potential target for anti-metastatic therapies [38]. In the progression of ovarian cancer, aberrant protein phosphorylation functions as a detrimental regulatory mechanism that significantly compromises the integrity and functionality of cellular signaling pathways.

#### ADP – ribosylation

ADP-ribosylation is a post-translational modification that couples a single unit of ADP-ribose (mono-ADP-ribosylation) or a polymer chain (poly-ADP-ribosylation) to a protein by the enzyme ADP-ribosyltransferase [40]. ADP-ribosylation also plays a crucial role in ovarian cancer research, with poly(ADP-ribose) polymerase 1 (PARP1) serving as the central enzyme [20]. PARP1 facilitates the poly-ADP-ribosylation of proteins by using nicotinamide adenine dinucleotide (NAD+) as a co-substrate [41]. When normal cells encounter DNA damage, PARP1 rapidly detects the damage site, recruits repair proteins by modifying histones, DNA polymerase, and other proteins through ADP-ribosylation, thereby initiating DNA damage repair processes and preserving genomic stability [42, 43]. In ovarian cancer, PARP1 exhibits a dual nature, acting as a “double-edged sword.” On one hand, ovarian cancer cells are characterized by rapid proliferation, intense metabolic activity, frequent DNA replication, and susceptibility to DNA damage [44]. The overexpression of PARP1 facilitates the efficient repair of damaged DNA in these cells, enabling them to evade apoptosis and sustain their proliferative capacity [45]. The study conducted by Hou Dong et al. demonstrated that PARP1 is extensively and highly expressed in ovarian cancer cell lines and tissue samples. Upon inhibition of PARP1 activity, cancer cell proliferation was significantly impeded, levels of reactive oxygen species (ROS) were elevated, and DNA double-strand breaks were exacerbated [46]. On the other hand, mild stress promotes PARP1 activation to initiate DNA repair without reducing NAD + levels [47]. Excessive activation of PARP1 depletes significant levels of NAD+, leading to an imbalance in intracellular energy metabolism and increased oxidative stress, which inhibits the growth of cancer cells [48]. Given the pivotal role of PARP1 in ovarian cancer, particularly in BRCA1/2 mutant ovarian cancer (where these cancer cells exhibit homologous recombination repair deficiencies and are more reliant on the PARP1-mediated single-strand repair pathway), PARP1 inhibitors such as olaparib and niraparib have emerged. By inhibiting the active site of PARP1, these inhibitors effectively interfere with ADP-ribosylation function, rendering DNA damage in cancer cells irreparable, thereby inducing apoptosis. This development has opened new avenues for precision treatment of ovarian cancer, especially in managing platinum-sensitive recurrent ovarian cancer, significantly prolonging patients’ progression-free survival and transforming clinical treatment paradigms.

## Analysis of mainstream Post-Modification targeted therapy strategies

### Clinical application of PARP inhibitors

Poly (ADP-ribose) polymerase (PARP) inhibitors are a milestone in the treatment of ovarian cancer, mainly including Olaparib, Niraparib and Rucaparib (Table 1). Olaparib represents the pioneering PARP inhibitor that has been approved by the FDA for the treatment of ovarian cancer harboring *BRCA* mutations [49]. Results from a cohort study demonstrated that patients with BRCA-like tumors experienced significantly longer progression-free survival (PFS) following olaparib treatment compared to placebo (36.4 months vs. 18.6 months; HR, 0.49; 95% CI, 0.37–0.65; *P* < 0.001) [50]. Another PARP inhibitor, nilaparib, also demonstrated significant efficacy in patients with platinum-sensitive relapsed ovarian cancer. According to a cohort study conducted by Gwynn Ison et al., patients with *BRCA* germline mutations who received nilaparib maintenance therapy exhibited a median PFS of 21.0 months, compared to 5.5 months for those receiving placebo. This represents a 73% reduction in the risk of disease recurrence or death [51]. Furthermore, clinical studies have demonstrated that drugs such as lucaparib and fluzoparib exhibit significant efficacy in treating ovarian cancer, either by prolonging PFS or by enhancing the objective response rate. Many clinical trials have shown improved survival in ovarian cancer patients using PARP inhibitors as maintenance therapy. PARP inhibitors have emerged as a cornerstone in the maintenance treatment of ovarian cancer, ushering in a new era of precision-targeted therapy for this disease.

**Table 1:** Comparison of key clinical data for PARP inhibitors in ovarian cancer

Name of drug	Indications for use	Clinical trial stage/type	Population of Patients	Median PFS/ORR
Olaparib	*BRCA*-mutated ovarian cancer	Phase III randomized controlled trial (SOLO-1)	Patients with newly diagnosed advanced ovarian cancer with a BRCA mutation	Median PFS following olaparib treatment compared to placebo (36.4 months vs. 18.6 months; HR, 0.49; 95%CI, 0.37–0.65; *P* < 0.001)[110].
	Platinum-sensitive recurrent ovarian cancer	Phase II/III combination chemotherapy trial	Patients with platinum-sensitive recurrent ovarian cancer	PFS was significantly longer with olaparib than with placebo (median, 8.4 months vs. 4.8 months from randomization on completion of chemotherapy; hazard ratio for progression or death, 0.35; 95% confidence interval [CI], 0.25 to 0.49; *P* < 0.001)[111].
Niraparib	Platinum-sensitive recurrent ovarian cancer	Phase III randomized controlled trial (NOVA)	Patients with *BRCA* germline mutations	Median PFS following niraparib treatment compared to placebo (21.0 months vs. 5.5 months, hazard ratio, 0.27; 95% confidence interval [CI], 0.17 to 0.41)[112].
	Non- *BRCA* -mutated ovarian cancer	Subgroup analysis was performed in the same trial	HRD-positive patients without *BRCA* mutations	The median follow-up for overall survival was 12.2 months (IQR 3.7–22.1). 13 (28%) of 47 patients in the primary efficacy population achieved an overall response according to RECIST (95% CI 15.6–42.6; one-sided *P* = 0.00053)[113].
Rucaparib	Platinum-sensitive recurrent ovarian cancer	Phase II clinical trial (ARIEL2)	Patients with *BRCA* mutations and homologous recombination deficiency (HRD)	The median duration of treatment for the 204 patients was 5.7 months (IQR 2.8–10.1). Median PFS after rucaparib treatment was 12.8 months (95% CI 9.0–14.7.7) in the BRCA mutant subgroup, 5.7 months (5.3–7.6) in the LOH high subgroup, and 5.2 months (3.6–5.5) in the LOH low subgroup[114].
	Advanced ovarian cancer	Phase III trial (ARIEL3)	Platinum-sensitive recurrent ovarian cancer regardless of *BRCA* status	Median PFS in patients with a BRCA-mutant carcinoma was 16.6 months (95% CI 13.4–22.9; 130 [35%] patients) in the rucaparib group versus 5.4 months (3.4–6.7; 66 [35%] patients) in the placebo group (hazard ratio 0.23 [95% CI 0.16–0.34]; *P* < 0.0001)[115].

Combining PTM-based drugs with immunotherapy, particularly PARP inhibitors, shows promise in boosting tumor immunogenicity and improving treatment results. PTMs are vital for protein regulation and immune responses. This integration can strengthen anti-tumor immunity by modulating the tumor microenvironment and enhancing tumor antigen presentation [52, 53]. PARP inhibitors enhance cancer treatment in tumors with homologous recombination deficiency by increasing DNA damage and immunogenic cell death, boosting tumor neoantigen presentation, and modulating the tumor microenvironment. This improves the effectiveness of immune checkpoint inhibitors like PD-1/PD-L1 or CTLA-4 blockers, especially in *BRCA*-mutated tumors [54]. Protein modifications like glycosylation, phosphorylation, and ubiquitination are crucial in regulating immune checkpoint molecules such as PD-L1 by influencing its stability, localization, and interactions, which are essential for tumor immune evasion. Targeting these modifications with small molecule inhibitors or monoclonal antibodies has demonstrated promising anti-tumor effects in preclinical studies, suggesting potential for clinical application [55, 56]. Overall, the integration of PTM-based therapies with immunotherapy represents a promising strategy to enhance antitumor immunity and overcome resistance mechanisms.

### Exploration of other potential targeted modification sites

#### Ubiquitination

**Fig. 1:**
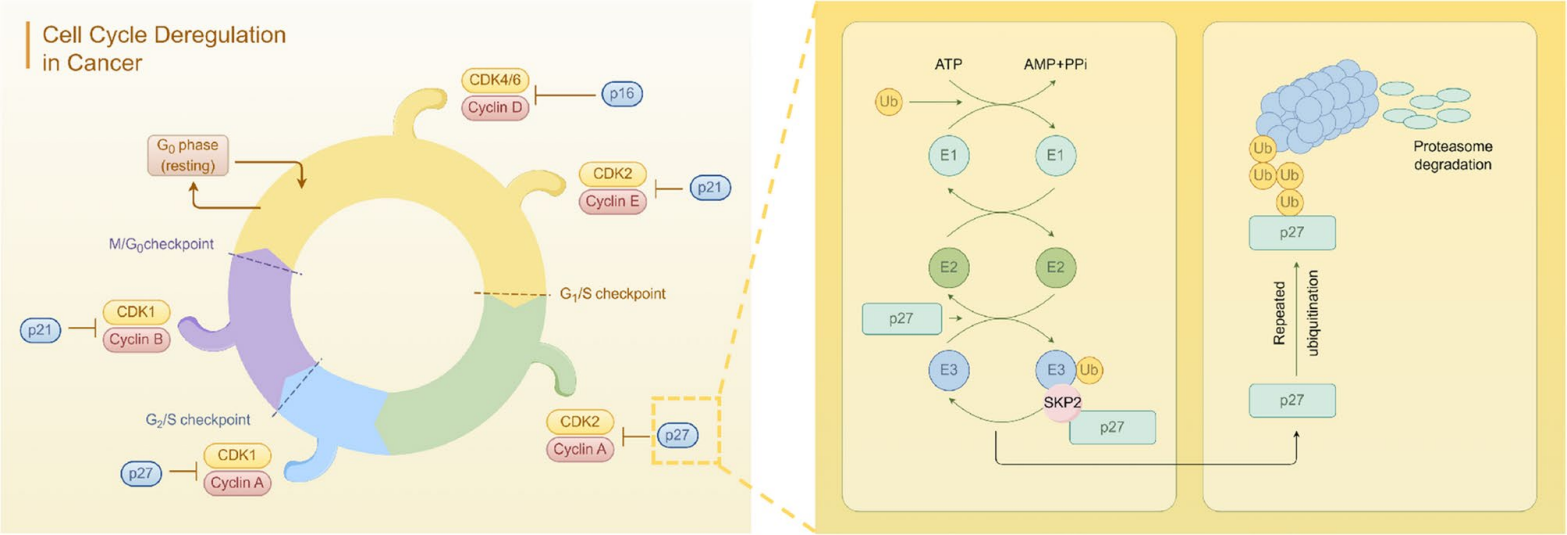
SKP2 acts as an E3 ubiquitin ligase to target and degrade p27 thereby disrupting cancer cell cycle control

Ubiquitination serves as the primary pathway for cellular protein degradation [57]. Ubiquitin activating enzyme (E1), ubiquitin conjugating enzyme (E2), and ubiquitin ligase (E3) collaboratively function to conjugate ubiquitin molecules onto target proteins. The ubiquitinated target proteins are subsequently recognized and degraded by the proteasome [58]. Dysregulation of genes, including mRNA, lncRNA, and miRNA, which are associated with ubiquitination-related enzymes, is frequently observed in the initiation and progression of various cancers, including ovarian cancer [59]. In ovarian cancer, the protein products of numerous oncogenes and tumor suppressor genes are subject to regulation through ubiquitination. For instance, SKP2, functioning as an E3 ubiquitin ligase, specifically targets the degradation of p27 (Fig. 1), a critical cell cycle-regulated tumor suppressor protein [60]. Elevated expression of SKP2 is frequently observed in ovarian cancer cells, where excessive ubiquitination results in the degradation of p27, thereby disrupting cell cycle control and promoting accelerated cancer cell proliferation [61, 62]. However, FBW7, an additional tumor-suppressing E3 ubiquitin ligase, is frequently mutated or downregulated in ovarian cancer. Consequently, oncogenic proteins such as c-Myc and Cyclin E, which should be targeted for degradation via ubiquitination, accumulate to significant levels, thereby promoting cancer cell proliferation [63]. Xu Fei et al. demonstrated that FBW7 interacts with the N6-methyladenosine (m6A) binding protein YTHDF2, promoting its ubiquitination and subsequent degradation. Overexpression of YTHDF2 globally modulates the m6A methylation levels of mRNA, leading to inhibition of pro-apoptotic gene BMF expression and facilitating ovarian cancer growth and invasion. Restoring FBW7 function or targeting YTHDF2 may offer a promising therapeutic strategy for ovarian cancer treatment [64]. Furthermore, deubiquitinating enzymes (DUBs) can reverse ubiquitination by removing the ubiquitin chain from target proteins [65]. Imbalances in the expression of certain DUBs, such as the overexpression of USP11 in ovarian cancer, contribute to chemotherapy resistance by stabilizing anti-apoptotic proteins like BIP [66]. Targeting USP11 may therefore provide a potential solution to overcoming chemotherapy resistance in ovarian cancer.

#### Methylation

DNA methylation typically occurs in the promoter regions of CPG-rich tumor suppressor genes and mediates gene silencing [67]. In ovarian cancer, the frequency of promoter methylation of the *RASSF1A* gene ranges from 30% to 50%. This gene encodes Ras effector proteins that play a critical role in regulating the cell cycle and inducing apoptosis. Methylation-induced silencing of *RASSF1A* contributes to the promotion of cancer cell proliferation and metastasis [68, 69]. Owing to the methylation of the DAPK promoter, DAPK expression is downregulated in the majority of cancers, thereby inhibiting the apoptosis pathway it mediates and facilitating tumor cell invasion [70, 71]. For example, the elevation in histone H3 trimethylation at lysine 27 (H3K27me3) is frequently associated with aberrant activation of the Polycomb Repressive Complex 2 (PRC2), which inhibits many tumor suppressor genes, resulting in enhanced dryness and impaired differentiation of ovarian cancer cells [72–74]. Liu Yi et al. discovered that the overexpression of PRMT1 in ovarian cancer tissues serves as a potential indicator of poor prognosis for patients with ovarian cancer. The underlying mechanism involves PRMT1-mediated methylation of BRD4 at residues R179, R181, and R183, which promotes BRD4 phosphorylation and consequently decreases ovarian cancer metastasis [75]. In addition, patients with early-stage EOC frequently present as asymptomatic or exhibit non-specific symptoms, underscoring the critical need for reliable diagnostic tools to facilitate early detection. A systematic review summarized and evaluated the performance of DNA methylation biomarkers derived from blood-based circulating tumor DNA (ctDNA) for the early diagnosis of EOC (Table 2). Among these, *RASSF1A*, *BRCA1*, and *OPCML* were the most extensively studied gene-specific methylation biomarkers, with *OPCML* demonstrating the most favorable performance metrics [76]. Currently, there is ongoing research and development in the field of DNA methylation, with demethylation drugs such as decitabine demonstrating the ability to partially reverse the methylation status of tumor suppressor genes.

**Table 2:** Application of methylation biomarkers in the early detection of ovarian cancer

Biomarkers	Related genes/pathways	Key Findings	References
OPCML	Tumor suppressor genes that regulate cell adhesion and signal transduction	Methylation levels were significantly increased in early ovarian cancer (stage I-II). Combined with CA125 can improve the accuracy of early diagnosis.	[76]
RASSF1A	Ras effector proteins that regulate cell cycle and apoptosis	RASSF1A promoter hypermethylation may be a useful molecular marker for early detection of ovarian tumors. RASSF1A promoter methylation was not correlated with the survival of patients with ovarian cancer.	[116, 117]
BRCA1	DNA repair and homologous recombination	Methylation cooperates with BRCA1 mutations to cause the HRD phenotype. Methylation status predicts PARP inhibitor sensitivity.	[118, 119]
HIC1	Transcriptional repressor that regulates Wnt/β-catenin	The methylation level was positively correlated with tumor stage.	[120, 121]

#### Palmitoylation

Palmitoylation is the reversible conjugation of fatty acids to cysteine ​​residues and affects a wide range of cellular processes, including protein trafficking, signal transduction, and immune responses [77, 78]. Dysregulated palmitoylation is associated with tumor progression, metastasis, and treatment resistance in various cancer types [79, 80]. Protein palmitoylation is a posttranslational modification involving the attachment of palmitate to a cysteine residue via S-palmitoylation or N-palmitoylation [81, 82]. A study demonstrated that the analysis of The Cancer Genome Atlas (TCGA) data on ovarian cancer demonstrated a significantly elevated expression of *ZDHHC12*. This gene exhibited the strongest positive correlation with the ROS pathway. When *ZDHHC12* was knocked out, this association was disrupted, leading to increased cellular complexity, ATP levels, mitochondrial activity, and both mitochondrial and cellular ROS production [83]. Furthermore, findings from an additional study revealed that ZDHHC12-mediated S-palmitoylation of CLDN3 significantly influences protein stability, subcellular localization, and tumorigenicity in ovarian cancer [84]. The findings of a study demonstrated that malate dehydrogenase 2 (MDH2), a pivotal enzyme in the tricarboxylic acid (TCA) cycle, undergoes palmitoylation at cysteine 138 (C138) residues, resulting in enhanced MDH2 activity. Inhibition of MDH2 both in vitro and in vivo leads to decreased mitochondrial respiration and reduced proliferation of ovarian cancer cells. Elevated levels of MDH2 palmitoylation observed in clinical samples from patients with high-grade serous ovarian cancer suggest that targeting ZDHHC18-mediated MDH2 palmitoylation may offer a promising therapeutic strategy for epithelial ovarian cancer (EOC) [85]. The findings suggest that palmitoyltransferase ZDHHC12/ZDHHC18 may serve as a promising therapeutic target for the treatment of ovarian cancer.

## Discussion

PTMs play a significant role in the development of chemotherapy resistance in ovarian cancer. These modifications can alter protein function, stability, localization, and interactions, thereby influencing cancer cell survival and response to treatment. One of the key PTMs involved in chemoresistance is phosphorylation, which can activate or deactivate signaling pathways that contribute to drug resistance. For instance, the phosphorylation of AKT and ERK1/2 has been implicated in enhancing autophagy and promoting resistance to cisplatin in ovarian cancer cells [86]. Hypermethylation of gene promoters, such as TGFBI, has been associated with paclitaxel resistance in ovarian cancer, suggesting that methylation status can influence drug sensitivity by altering gene expression [87]. Similarly, LAMA3 hypermethylation has been linked to chemotherapy resistance and poor prognosis in ovarian cancer, indicating that methylation patterns can serve as potential biomarkers for treatment outcomes [88].

The emergence of resistance to PARP inhibitors is posing a significant challenge in the treatment of ovarian cancer. During the process of cancer treatment, cancer stem cells show the characteristics of quiescence, strong DNA repair ability, and strong self-renewal ability [89]. In *BRCA1/2* mutant ovarian cancer cells that depend on PARP-mediated single-strand repair due to homologous recombination repair (HR) deficiency caused by *BRCA* gene mutations, a subset of these cells may acquire secondary reversion mutations in the *BRCA1/2* genes. These reversion mutations can restore partial or complete HR functionality, enabling precise DNA damage repair and allowing cells to evade the effects of PARP inhibitors [90]. A clinical study investigating cfDNA testing in patients who experienced disease progression following treatment with PARP inhibitors revealed that approximately 21% of ovarian cancer patients harbored *BRCA*-reversing mutations [91]. When PARP activity is inhibited, alternative DNA repair mechanisms within the cell, including non-homologous end joining (NHEJ) and the Fanconi anemia pathway, become aberrantly activated to compensate for the impaired DNA repair [92]. The up-regulated expression of Ku70/80 and other key proteins involved in the NHEJ pathway accelerates the repair of DNA breaks induced by PARP inhibitors [93–95]. This mechanism contributes to maintaining genomic stability in cancer cells, thereby significantly reducing the efficacy of PARP inhibitors, increasing the risk of disease recurrence, limiting subsequent therapeutic options, and leading to a poorer prognosis.

Several post-translationally modified drugs exhibit suboptimal target specificity. Several ubiquitin-modified targeted drugs are engineered to specifically modulate the activity of particular E3 ubiquitin ligases or deubiquitinating enzymes [96]. However, the intracellular ubiquitination system is highly complex, encompassing a diverse array of enzymes with overlapping functionalities. When pharmaceutical agents intervene, in addition to targeting the intended proteins, they may inadvertently interact with other enzymes of similar structure, thereby disrupting normal intracellular protein homeostasis [97]. For instance, inhibitors that target a carcinogenic E3 ligase may also affect another E3 ligase involved in the regulation of normal cellular metabolism due to structural similarities. While these inhibitors exhibit some efficacy against cancer cells, they can concurrently induce metabolic disorders in healthy cells and adverse reactions such as hepatic dysfunction and gastrointestinal discomfort [98]. These side effects not only compromise patients’ quality of life but may necessitate dose reduction or discontinuation of treatment, thereby impeding the therapeutic process and potentially diminishing long-term efficacy.

In the treatment of ovarian cancer, combination therapy has emerged as a promising approach, offering new hope to overcome the challenges. A multicenter Phase II trial enrolled patients with platinum-sensitive recurrent ovarian cancer and randomized them to receive either the combination of the PARP inhibitor olaparib with paclitaxel and carboplatin or chemotherapy alone. The results demonstrated that the PFS was significantly prolonged in the combination treatment group, with a median PFS of 12.2 months compared to 9.6 months in the chemotherapy-alone group. This effect is attributed to the mechanism whereby chemotherapy induces extensive DNA damage, leading cancer cells to rely on PARP for repair [99]. The inhibition of PARP by olaparib blocks this repair pathway, resulting in the accumulation of DNA damage and enhanced apoptosis, thereby creating a synergistic anticancer effect.

The emergence of immunotherapy has invigorated combination strategies, introducing novel opportunities for synergistic treatments. Specifically, immunotherapy as adjuvant therapy, combined with chemotherapy, radiotherapy, use of anti-angiogenic drugs and PARP inhibitors, can significantly improve the efficiency of ovarian cancer treatment [100]. PARP inhibitors induce DNA damage in cancer cells, leading to the release of tumor-associated antigens and activation of the immune response [101]. Meanwhile, immune checkpoint inhibitors alleviate immune suppression and augment T cell-mediated cytotoxicity against cancer cells [102]. Preclinical studies have demonstrated that the combination regimen markedly inhibits tumor growth and extends survival in a murine model of ovarian cancer [103]. Additionally, sustained remissions have been observed in certain patients during clinical trials [104].

Proteolysis-targeting chimeras (PROTACs) have emerged as a promising therapeutic strategy in the treatment of ovarian cancer [105]. PROTACs are a groundbreaking technology for targeted protein degradation, using the ubiquitin-proteasome system to selectively degrade specific proteins. This offers a novel method for modulating protein levels post-translationally, aiding in biological research and therapeutic applications [106]. This evolution marks a new era in drug development, particularly in the realm of targeted protein degradation (TPD), where PROTACs are now being used to target previously ‘undruggable’ proteins and recruit new E3 ligases, thus expanding the scope of druggable targets [107]. A study found that two new PROTAC compounds outperform the FDA-approved drug brigatinib in inhibiting ovarian cancer cell motility by degrading various oncogenic FER fusion proteins [108]. The widespread presence of E3 ligases in both cancerous and healthy tissues poses a challenge due to on-target toxicities. Targeting E3 ligases that are abundant in tumors but scarce in normal tissues could lead to the creation of tumor-specific PROTACs, improving their therapeutic effectiveness [109]. In summary, PROTACs represent a transformative approach in cancer therapy, particularly in ovarian cancer, by offering a novel mechanism for targeted protein degradation.

## Conclusion and prospect

**Fig. 2:**
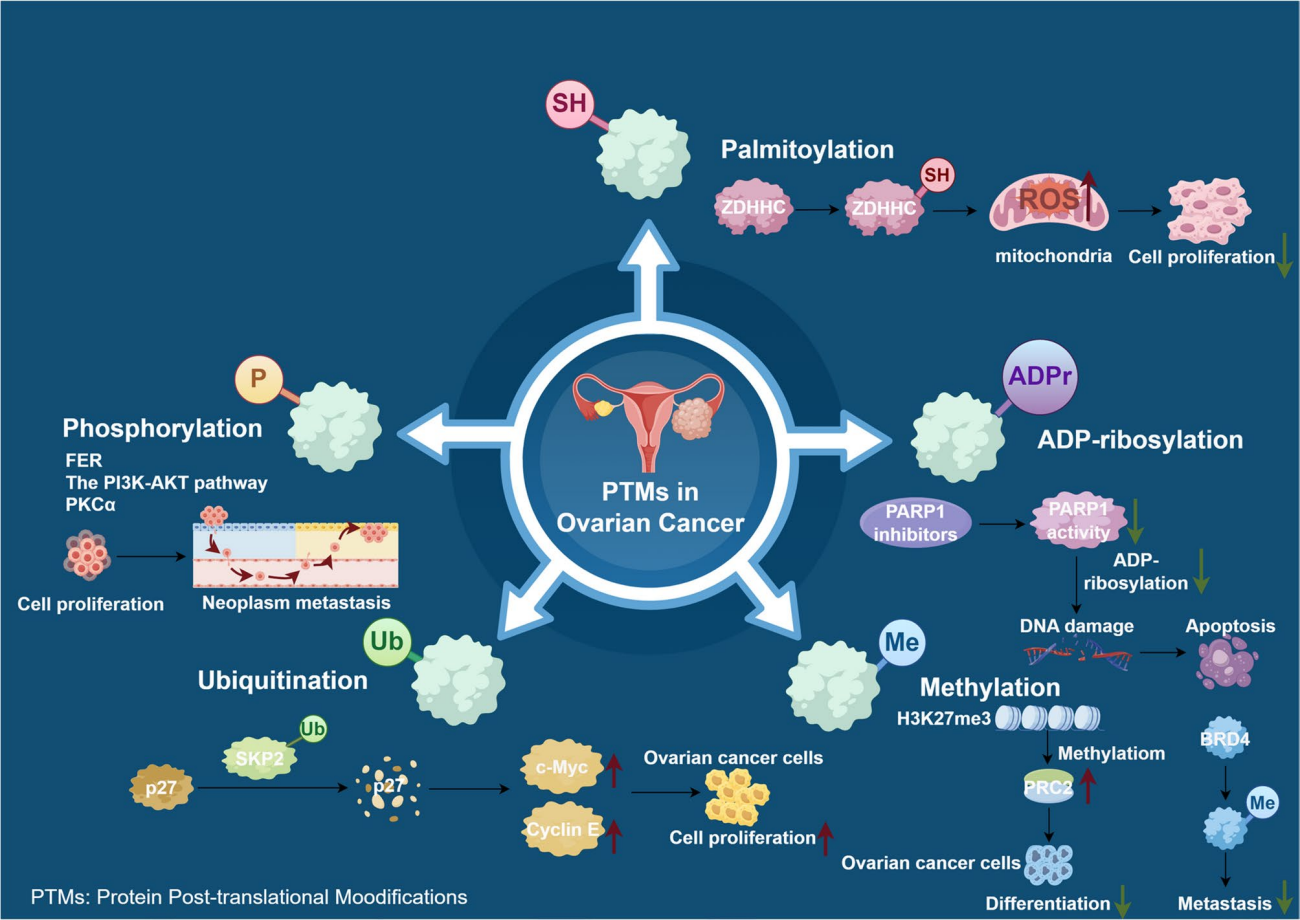
Pathway map of different PTMs in ovarian cancer

This study delves deeply into the crucial role of post-translational modifications in the pathogenesis and treatment of ovarian cancer, revealing their significant potential as therapeutic targets (Fig. 2). The pathogenesis of ovarian cancer is complex, involving genetic factors such as *BRCA1/2* gene mutations, as well as the synergistic effects of hormones and environmental factors, which promote the proliferation of cancer cells. Post-translational modifications play a key bridging role in this process, with abnormal modifications such as phosphorylation and ADP-ribosylation disrupting cellular signaling and DNA repair, thereby facilitating tumor development. PARP inhibitors, based on the principle of “synthetic lethality,” have shown remarkable efficacy in *BRCA*-mutated ovarian cancer, with several drugs significantly extending the progression-free survival of patients, opening a new chapter in precision treatment. The exploration of other modification targets such as ubiquitination and methylation broadens the horizons for targeted therapy, although challenges remain, the potential is immense. Future research should aim to identify ZDHHC enzyme inhibitors, like small molecules or mimetic peptides, and assess their impact on mitochondrial metabolism and ROS pathways. Combining these inhibitors with metabolic modulators, such as MDH2 or PARP inhibitors, could help overcome resistance through synergistic effects. Targeting DUB overexpression, like USP11, with covalent inhibitors or PROTAC degraders may stabilize pro-apoptotic proteins and restore chemotherapy sensitivity. Single-cell multi-omics can analyze DUB expression in tumor heterogeneity and identify predictive markers, such as USP11 activity, for precision medicine. Additionally, integrating demethylating agents like decitabine with PRMT inhibitors (targeting PRMT1) could reverse tumor suppressor gene silencing and inhibit metastasis pathways. Investigate combining PTM-targeted drugs (e.g., PARP inhibitors) with immune checkpoint inhibitors to boost T cell responses by releasing tumor antigens (Table 3). Challenges include the conserved catalytic domains of PTM enzymes (like kinases and DUBs), which make small molecule inhibitors likely to disrupt normal cell functions (e.g., ZDHHC inhibitors affecting neuronal palmitylation). Additionally, tumor heterogeneity, compensatory mechanisms, and a lack of biomarkers pose significant hurdles.

**Table 3:** Advances in the molecular mechanism and related drugs of major PTMS in ovarian cancer

PTM type	Molecular mechanism	Relevant drugs/treatment strategies
Phosphorylation	Activation of PI3K-AKT signaling (FER phosphorylation of IRS4).	Small molecule inhibitors targeting kinases, such as PI3K/AKT/mTOR inhibitors, MEK inhibitors, etc.
	Activation of MAPK pathway (ERK phosphorylation).	
	PKCα phosphorylates downstream target proteins and enhances cancer cell migration and invasion.	
ADP-ribosylation	PARP1 mediates DNA damage repair and maintains genome stability.	PARP Inhibitors: Olaparib, Niraparib, Rucaparib
	*BRCA*-mutated cancer cells rely on the single-strand repair pathway of PARP1.	
Ubiquitination	Overexpression of SKP2 degrades p27 and promotes proliferation.	E3 ubiquitin ligase inhibitors (e.g., small molecules targeting SKP2 or FBW7)
	Inactivation of FBW7 leads to accumulation of c-Myc.	DUB inhibitors (e.g., compounds targeting USP11)
	USP11 overexpression of stable anti-apoptotic proteins, such as BIP, leads to chemotherapy resistance.	PROTAC technology degrades specific target proteins
Methylation	Promoter methylation of RASSF1A, DAPK and other genes results in silencing.	Demethylating Agent: Decitabine
	PRMT1 mediates BRD4 methylation to promote metastasis.	PRMT inhibitors (e.g., compounds that target PRMT1)
	Aberrant methylation of PRC2 represses tumor suppressor genes.	DNA methylation markers, such as OPCML, are used for early diagnosis
Palmitoylation	ZDHHC12/18 mediates palmitoylation of CLDN3 and MDH2 to enhance protein stability and cancer-promoting activity.	ZDHHC enzyme inhibitors (e.g., small molecules or mimetic peptides targeting ZDHHC12/18)
	Regulation of mitochondrial metabolism and ROS production.	Inhibition of MDH2 activity (e.g., metabolic modulators)

## Data Availability

No datasets were generated or analysed during the current study.

## Abbreviations

PTMs: Post-translational modifications
HGSC: High-grade serous carcinoma
OC: Ovarian cancer
CDKs: Cyclin-dependent kinasesii
PKMTs: Protein lysine methyltransferases
PRMTs: Protein arginine methyltransferases
KATs: Lysine acetyltransferases
KDACs: Lysine deacetylases
ARTs: ADP-ribosyltransferases
FDA: Food and Drug Administration
PARP: Poly-ADP-ribose polymerase
HBOC: Hereditary breast and ovarian cancer
EMT: Epithelial–mesenchymal transition
ES-SSL-OXA/PTX: Estrogen-targeted polyethylene glycol liposome
EOC: Epithelial ovarian cancer
NSCLC: Non-small cell lung cancer
PI3K: Phosphatidylinositol 3-kinase
AKT: Protein kinase B
IRS4: Insulin receptor substrate 4
MAPK: Mitogen-activated protein kinase
FER: Non-receptor tyrosine kinase
ERK: Extracellular regulated kinase
PKC: Protein kinase C
ROS: Reactive oxygen species
NAD+: Nicotinamide adenine dinucleotide
PFS: Progression-free survival
DUBs: Deubiquitinating enzymes
PRC2: Polycomb Repressive Complex 2
TCGA: The Cancer Genome Atlas
MDH2: Malate dehydrogenase 2
HR: Homologous recombination repair
NHEJ: Non-homologous end joining
PROTACs: Proteolysis-targeting chimeras
